# Elevated glucocorticoid concentrations during gestation predict reduced reproductive success in subordinate female banded mongooses

**DOI:** 10.1098/rsbl.2015.0620

**Published:** 2015-10

**Authors:** J. L. Sanderson, H. J. Nichols, H. H. Marshall, E. I. K. Vitikainen, F. J. Thompson, S. L. Walker, M. A. Cant, A. J. Young

**Affiliations:** 1 Centre for Ecology and Conservation, University of Exeter (Penryn Campus), Penryn TR10 9FE, UK; 2 School of Natural Science and Psychology, Liverpool John Moores University, Liverpool L3 3AF, UK; 3 Chester Zoo Wildlife Endocrinology Laboratory, Caughall Road, Upton-by-Chester, Chester CH2 1LH, UK

**Keywords:** reproductive conflict, intra-sexual selection, female competition, cooperative breeding

## Abstract

Dominant females in social species have been hypothesized to reduce the reproductive success of their subordinates by inducing elevated circulating glucocorticoid (GC) concentrations. However, this ‘stress-related suppression' hypothesis has received little support in cooperatively breeding species, despite evident reproductive skews among females. We tested this hypothesis in the banded mongoose (*Mungos mungo*), a cooperative mammal in which multiple females conceive and carry to term in each communal breeding attempt. As predicted, lower ranked females had lower reproductive success, even among females that carried to term. While there were no rank-related differences in faecal glucocorticoid (fGC) concentrations prior to gestation or in the first trimester, lower ranked females had significantly higher fGC concentrations than higher ranked females in the second and third trimesters. Finally, females with higher fGC concentrations during the third trimester lost a greater proportion of their gestated young prior to their emergence from the burrow. Together, our results are consistent with a role for rank-related maternal stress in generating reproductive skew among females in this cooperative breeder. While studies of reproductive skew frequently consider the possibility that rank-related stress reduces the conception rates of subordinates, our findings highlight the possibility of detrimental effects on reproductive outcomes even after pregnancies have become established.

## 1. Introduction

In animal societies, subordinate females often have lower reproductive success than dominant females. The stress-related suppression hypothesis proposes that dominant females suppress subordinate reproduction through behaviours that lead to chronic elevations in circulating glucocorticoids (GCs) and consequent reproductive downregulation [1–4]. Notably though, compelling support for this hypothesis remains scarce in cooperatively breeding societies, where reproductive skews among females are frequently apparent ([1,2]; but see [3,5]). Stress-related suppression might only be necessary, however, in the subset of cooperative breeders in which subordinate females do still attempt to breed, as complete reproductive restraint by subordinates might otherwise obviate the need for dominants to stress their subordinates [3,6,7]. Furthermore, stress-related suppression could actually be difficult to detect using the approach most-commonly employed to test it (comparisons of the average GC levels of dominants and subordinates), if dominants target only a subset of likely breeders and do so only during periods when subordinate reproduction would otherwise be costly to dominants [3,5,6]. These suggestions have led to calls for further tests in cooperatively breeding species in which subordinates do attempt to breed, focusing on those subordinates attempting to breed at the same time as their dominants [3,6].

While socially induced GC elevations have frequently been considered a potential cause of reduced conception rates among subordinates, they also have the potential to compromise the outcomes of established pregnancies. For example, elevated GCs during pregnancy may impact *in utero* or early post-natal development and affect offspring health, condition and survival [6,7]. While studies of cooperatively breeding mammals have shown that being subjected to aggression by the dominant female is associated with increased abortion rates among subordinates [3,8], whether rank-related maternal stress compromises reproductive outcomes among subordinates that do manage to carry to term has yet to be investigated. If subordinate reproductive success was reduced as a result of elevated GC concentrations during gestation, then one might make three predictions: pregnant females of lower social rank will have (i) reduced reproductive success and (ii) elevated GC concentrations during gestation, and (iii) females experiencing higher gestational GCs will have reduced reproductive success.

Here, we test these three predictions with a detailed investigation of faecal glucocorticoid (fGC) concentrations and reproductive success in female banded mongooses (*Mungos mungo*). Banded mongooses live in stable cooperatively breeding groups comprising a ‘core’ of breeding adults (one to five females and three to seven males) that reproduce three to four times per year, alongside a subset of younger individuals that breed occasionally [9]. Aggression received by pregnant subordinates can result in eviction and abortion [8], but pregnant subordinates do often breed successfully alongside pregnant dominants [9]. The rank-related patterns of reproductive success among females that carry to term have yet to be investigated, along with the role that GCs may play in generating them.

## 2. Material and methods

We studied a population of banded mongooses living in Queen Elizabeth National Park, Uganda (0°12′ S; 29°53′ E) between December 2010 and April 2014. All animals were marked and habituated to close observation (less than 5 m). Groups were observed every 1–4 days to record all breeding events. We ran generalized linear mixed models (GLMMs) using the lme4 package [10] in R v. 3.1.1 [11] with Poisson and binomial data fitted with log and logit link functions, respectively. Female, social group and litter identities were included as random intercepts in all models to control for repeated measures.

Pregnancy can be detected at around 40 days by swelling of the abdomen [12] and birth can be detected by a sudden decrease in female body size [13]. Females were captured during pregnancy to estimate the number of fetuses each carried by palpation [12]. We assigned maternity using a combination of phenotypic and microsatellite data; full details are given in [14]. Analyses of reproductive success were limited to communal litters in which at least one pup emerged.

We collected 218 faecal samples from 35 females prior to and during gestation (2.5 ± 0.3 samples per female pregnancy, mean ± s.e.; number of samples collected per time period: pre-gestation = 59 samples, first trimester = 57 samples, second trimester = 45 samples, third trimester = 54 samples). Full details of sample collection and hormone analysis including validations are given in [15]. In brief, all samples were collected between 06.30 and 10.00 and stored on ice [15]. Hormones were extracted from faecal samples using a wet-weight extraction (adapted from [16]) and then analysed using an enzyme immunoassay.

### (a) Do lower ranking females experience reduced reproductive success?

We calculated three measures of reproductive success for each female recorded as having given birth: (i) the number of fetuses, (ii) the number of emergent offspring, and (iii) the proportion of fetuses surviving to emergence. We fitted each of these three measures as a response variable in GLMMs. Rank (determined by ranked age following [17]) was fitted as a fixed effect in each model as were female age, group size, rainfall (month prior to conception) and pre-conception body mass [13] to control for other factors which may lead to variation in reproductive success.

### (b) Do lower ranking females experience elevated faecal glucocorticoids during gestation?

We fitted fGC concentrations as a response variable in a GLMM with rank as the main predictor of interest. As GC concentrations may vary within a breeding attempt, we also fitted an interaction between rank and stage of pregnancy (pre-gestation, first trimester, second trimester, third trimester) as well as fixed effects of female age, group size, rainfall and pre-conception body mass to control for other factors which may contribute to fGC variation.

### (c) Do females with higher faecal glucocorticoids during gestation have reduced reproductive success?

We fitted the number of emergent offspring and the proportion of fetuses surviving to emergence as response variables in two separate GLMMs with fGCs during the third trimester as the predictor of interest. We focused this analysis on fGCs in the third trimester because that is when we saw the clearest difference in fGCs between low- and high-ranking females.

## 3. Results

**Figure 1.**
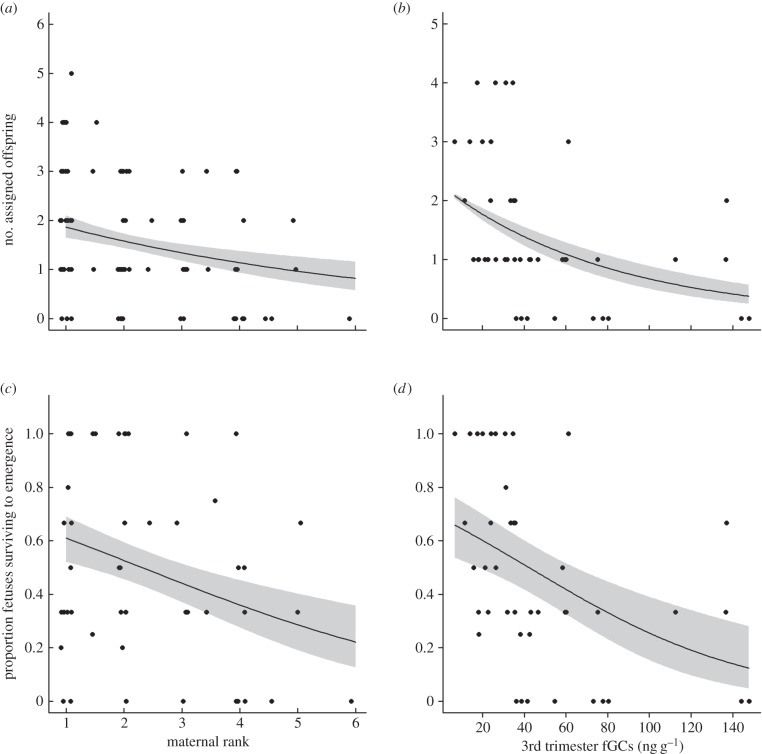
(*a*,*c*) Maternal rank and (*b*,*d*) gestational fGC concentrations predict female reproductive success. Points show raw values and lines with shaded regions show predicted trends with confidence intervals from GLMMs.

**Figure 2.**
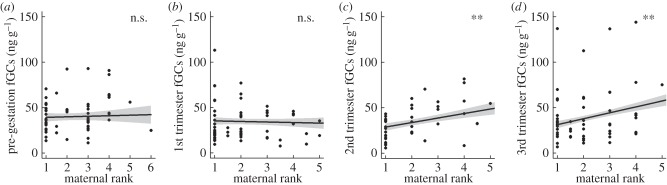
Female fGC concentrations vary during gestation dependant on maternal rank. Dots show raw values and lines and shaded areas show predicted estimates and confidence intervals from a GLMM. Significance values from post-hoc testing of the effect of maternal rank on fGC concentrations: (*a*) within a pre-gestation phase and (*b*–*d*) during three trimesters where n.s. *p* > 0.05; **p < 0.001.

Lower ranking females that carried to term experienced lower reproductive success than higher ranking females, both when measured as the number of assigned offspring (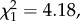 *p* = 0.041; figure 1*a*) and the proportion of fetuses surviving to emergence (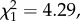 *p* = 0.038; figure 1*c*). There was no effect of rank on the number of fetuses carried by a female (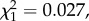 *p* = 0.87). We found a significant interaction between female rank and pregnancy stage on fGC concentrations: lower ranking females did not differ from higher ranking females prior to conception or during the first trimester but had elevated fGCs during the second and third trimesters (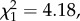 *p* = 0.041; figure 2). Females experiencing higher fGC concentrations during the third trimester had fewer assigned offspring than those with lower GCs (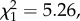 *p* = 0.022; figure 1*b*) and a lower proportion of their fetuses survived to emergence (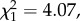 *p* = 0.044; figure 1*d*). Full model outputs are included in the electronic supplementary material S1.

## 4. Discussion

Our findings are consistent with the hypothesis that subordinate female banded mongooses exhibit reduced reproductive success as a result of rank-related maternal stress during gestation. Lower ranked females had lower reproductive success than higher ranked females (despite conceiving litters of the same size), both when measured as the proportion of fetuses surviving to emergence and the number of emergent offspring. Although higher and lower ranked females had similar fGC concentrations prior to gestation and during the first trimester, lower ranked females showed significantly elevated fGC concentrations during the second and third trimesters. These results highlight the possibility that stress-related suppression of subordinate reproduction arises through gestational effects that compromise offspring survival either during the latter stages of pregnancy or soon after birth (prior to emergence from the burrow). Accordingly, females that experienced higher fGC concentrations during the third trimester had fewer emergent pups and a lower proportion of fetuses surviving to emergence.

Rank-related differences in reproductive success among female mammals commonly occur due to differences in conception rates, either because subordinate females exercise reproductive restraint or because their ability to conceive is compromised by active interference by dominant females [18,19]. By contrast, we have demonstrated a rank-related difference in reproductive success within females that carry to term. As there was no observable rank-related variation in litter size *in utero*, this rank-related difference in reproductive success could well have arisen from pre-natal developmental impacts on offspring survival either during late pregnancy or during the early post-natal period. A role for rank-related maternal stress during late gestation in generating these effects on offspring survival would be consistent with experimental evidence that late-gestational GC elevations can inhibit offspring development [4,20]. In the absence of experimental evidence of a role for maternal GC elevations, however, it is also possible that alternative mechanisms, such as early post-natal infanticide [21], play a role in generating the observed rank-related variation in offspring survival from detection as a fetus to emergence from the burrow.

The stress-related suppression hypothesis posits that elevated GC concentrations observed in lower ranking females are a result of aggression from dominant females. However, conspicuous aggression among female banded mongooses is rare outside of eviction events [9]. As such, the elevated GC concentrations observed here may not be a product of overt aggression. Our findings cannot be attributed instead to simple age effects, in which younger females struggle to meet the resource-demands of gestation (and hence exhibit differential GC elevations), as our analyses control for variation in absolute age and attribute variation in both reproductive success and gestational GC concentrations to variation in rank *per se*. However, the gestational GC elevations of lower ranked females could arise at least in part from energetic differences during gestation. For example, subordinates may be competitively excluded from resources by dominant females. Alternatively, as intra-sexual conflict among females may frequently be resolved without overt physical conflict, these GC elevations could also reflect responses to more subtle rank-related outcomes, such as social isolation [22]. Either way, our findings highlight the possibility that stress-related suppression of subordinate reproduction may occur in the absence of conspicuous aggression.

## Supplementary Material

Model outputs and sample sizes

## Supplementary Material

Data used in study

## Data accessibility

All data analysed in this study are available in the electronic supplementary material S2.
