# Supplementary material for: Elevated glucocorticoid concentrations during gestation predict reduced reproductive success in subordinate female banded mongooses
Source: Biol Lett. 2015 Oct;11(10):20150620. doi: 10.1098/rsbl.2015.0620 (PMC4650177; doi:10.1098/rsbl.2015.0620)
Supplement: Model outputs and sample sizes [file rsbl20150620supp1.docx]

Supplementary Material 1: Model outputs and sample sizes

| **Table SI1 Factors effecting female reproductive success when measured per breeding attempt. Significant results are given in bold.** | | | | | | | | | | | | | | | |
| --- | --- | --- | --- | --- | --- | --- | --- | --- | --- | --- | --- | --- | --- | --- | --- |
|  | Number of foetuses carried by a female | | | | | Number of emergent offspring assigned to a female | | | | | Proportion of foetuses per female that survive to emergence | | | | |
| Explanatory terms | Effect Size ± SE | | | χ^2^ | P | Effect Size ± SE | | | χ^2^ | P | Effect Size ± SE | | | χ^2^ | P |
|  |  |  |  |  |  |  |  |  |  |  |  |  |  |  |  |
| Social rank | -0.01 | ± | 0.06 | 0.027 | 0.87 | **-0.16** | **±** | **0.08** | **4.18** | **0.041** | **-0.34** | **±** | **0.17** | **4.29** | **0.038** |
| Age (years) | 0.03 | ± | 0.05 | 0.36 | 0.55 | 0.04 | ± | 0.05 | 0.60 | 0.44 | -0.02 | ± | 0.20 | 0.02 | 0.88 |
| Pre-conception body mass (g) | 0.00 | ± | 0.00 | 0.51 | 0.47 | **0.0009** | **±** | **0.0005** | **4.37** | **0.037** | 0.00 | ± | 0.00 | 1.72 | 0.19 |
| Rainfall | 0.13 | ± | 0.19 | 0.48 | 0.49 | 0.006 | ± | 0.24 | <0.001 | 0.98 | -0.93 | ± | 0.51 | 3.48 | 0.062 |
| Group size | 0.00 | ± | 0.00 | 0.00 | 0.99 | **-0.03** | **±** | **0.02** | **4.19** | **0.041** | -0.02 | ± | 0.04 | 0.12 | 0.73 |
| Constant | 1.08 | ± | 0.08 |  |  | 0.15 | ± | 0.72 |  |  | 0.79 | ± | 0.49 |  |  |
|  | 49 observations from 25 females in 21 litters and 4 packs. Coefficients given on log scale. | | | | | 82 observations from 34 females in 36 litters and 5 packs. Coefficients given on log scale. | | | | | 49 observations from 25 females in 21 litters and 4 packs. Coefficients given on logit scale. | | | | |

| **Table S1.2 Factors affecting the fGC concentrations prior to and during gestation** | | | | | | |  | |
| --- | --- | --- | --- | --- | --- | --- | --- | --- |
| **Explanatory terms** | | **Effect Size ± SE** | | | **χ^2^** | **p** |  |  |
|  | |  | | |  |  |  |  |
| **Social rank * trimester** | **1st** | **-5.11** | **±** | **3.21** | **10.94** | **0.012** |  |  |
|  | **2nd** | **4.46** | **±** | **3.39** |  |  |  |  |
|  | **3rd** | **4.39** | **±** | **3.39** |  |  |  |  |
| Social rank | | 1.85 | ± | 2.25 | n/a | n/a |  |  |
| Trimester | 1st | 7.07 | ± | 8.07 | n/a | n/a |  |  |
|  | 2nd | -13.88 | ± | 8.53 |  |  |  |  |
|  | 3rd | -5.60 | ± | 8.51 |  |  |  |  |
| Age (years) | | -0.67 | ± | 1.31 | 0.38 | 0.54 |  |  |
| Pre-conception body mass (g) | | 0.02 | ± | 0.01 | 2.38 | 0.12 |  |  |
| Rainfall | | 1.38 | ± | 5.53 | 0.07 | 0.79 |  |  |
| Group size | | 0.33 | ± | 0.39 | 0.86 | 0.36 |  |  |
| Constant | | 31.92 | ± | 6.40 |  |  |  |  |
| 215 observations from 35 females in 41 litters and 5 packs. | | | | | | |  |  |
